# Influence of Fracture Width on Sealability in High-Strength and Ultra-Low-Permeability Concrete in Seawater

**DOI:** 10.3390/ma6072578

**Published:** 2013-06-25

**Authors:** Daisuke Fukuda, Yoshitaka Nara, Daisuke Hayashi, Hideo Ogawa, Katsuhiko Kaneko

**Affiliations:** 1 Faculty of Engineering, Hokkaido University, Kita 13 Nishi 8, Kita-ku, Sapporo, Hokkaido 060-8628, Japan; E-Mail: kaneko@geo-er.eng.hokudai.ac.jp; 2 Graduate School of Engineering, Kyoto University, Kyoto Daigaku Katsura, Nishikyo-ku, Kyoto 615-8540, Japan; E-Mail: nara.yoshitaka.2n@kyoto-u.ac.jp; 3 Taiheiyo Consultant Co., Ltd., Ohsaku, Sakura, Chiba 285-8655, Japan; E-Mails: d-hayashi@rwmc.or.jp (D.H.); hideo_ogawa@taiheiyo-c.co.jp (H.O.)

**Keywords:** fracture, self-sealing, micro-focus X-ray CT, image subtraction, high-strength and ultra-low-permeability concrete, radioactive waste disposal

## Abstract

For cementitious composites and materials, the sealing of fractures can occur in water by the precipitation of calcium compounds. In this study, the sealing behavior in a macro-fractured high-strength and ultra-low-permeability concrete (HSULPC) specimen was investigated in simulated seawater using micro-focus X-ray computed tomography (CT). In particular, the influence of fracture width (0.10 and 0.25 mm) on fracture sealing was investigated. Precipitation occurred mainly at the outermost parts of the fractured surface of the specimen for both fracture widths. While significant sealing was observed for the fracture width of 0.10 mm, sealing was not attained for the fracture width of 0.25 mm within the observation period (49 days). Examination of the sealed regions on the macro-fracture was performed using a three-dimensional image registration technique and applying image subtraction between the CT images of the HSULPC specimen before and after maintaining the specimen in simulated seawater. The temporal change of the sealing deposits for the fracture width of 0.10 mm was much larger than that for the fracture width of 0.25 mm. Therefore, it is concluded that the sealability of the fracture in the HSULPC is affected by the fracture width.

## 1. Introduction

For the geological disposal of radioactive waste, the radioactive intensity of radionuclides can be reduced by engineered barriers, such as bentonite buffers, and natural barriers, such as rock mass. If a repository of radioactive waste is located in an area where the hydraulic gradient and permeability are high, the retardation of radionuclide migration by these barriers may not be sufficient. To retard radionuclide migration, several alternative concepts of radioactive waste packages are being developed. For instance, high-strength and ultra-low-permeability concrete (HSULPC) is planned for radioactive waste and the geological disposal of transuranic (TRU) waste [1,2,3] to confine radionuclides with low adsorption by engineered barriers, such as the ^14^C included in the TRU waste in Japan.

Generally, water migrates through networks of cracks and pores in a solid. In cementitious composites and materials, it has been reported that this water migration may (a) result in the formation of calcium carbonate or calcium hydroxide; (b) block cracks by impurities in the water and loosen concrete particles from crack spalling; (c) further hydrate the unreacted cement or cementitious materials; (d) and expand the hydrated cementitious matrix in the crack flanks (swelling of C–S–H) [4]. In case that age of concrete is sufficiently old, the primary mechanism could be the precipitation of calcium carbonate [5,6,7,8,9].

A recent paper on concrete [10] showed that water permeability increases significantly with increasing cracking. Thus, the sealing of cracks and pores may occur and affect permeability. This phenomenon has been investigated by various researchers (e.g., [5,6,7,8,9,10,11,12,13,14,15,16,17]). Edvardsen [5] showed that the sealing of a crack occurs by the precipitation of calcium carbonate, generated from CO_3_^2−^ in water and Ca^2+^ in the cement paste. The fracture width and applied water pressure affect the sealing significantly, but the composition of the surrounding water has little effect [5]. Reinhardt and Jooss [14] investigated the temperature dependence of the sealing and showed that sealing is enhanced with increasing temperature, up to 80 °C. Yang* et al.* [9] showed that sealing occurs if the crack width is less than 0.15 mm. 

The crack-sealing was also observed in various rocks and there is quite a large volume of literature on fracture sealing in rock. In terms of sealing of fracture in rock fault, Gratier* et al.* [18] and Evans and Chester [19] reported that the vein minerals related to active faults are always sealed by nearby solution cleavage and stylolites in the country rocks. Fisher and Brantley [20] reported that the sealing of rock is observed due to local migration of silica from the rock matrix to veins with an approximate balance between the silica dissolved from the rock matrix and the amount of quartz precipitated in veins. Gratier* et al.* [18] also reported that, within the fault zone where the rapid slip caused an increase of the overall permeability and reduced the fluid pressure to levels approaching hydrostatic values, fluid/rock interactions occurs on the free faces produced by earthquake and is characterized by fast kinetics triggering the self-healing of fractured minerals. The time scales of such crack-sealing, controlled by the kinetics of pressure solution and associated with deposition processes, are on the order of several tens of years to several million years and are strongly dependent on temperature and the rock texture. In addition, self-sealing of various rock types was observed in the excavation damaged zone (EDZ) and Tsang* et al.* [21] discussed the crack-sealing in rock from various aspects such as permeability reduction and temperature. It was reported that self-sealing was not expected in crystalline rocks. For salt, healing of the EDZ can be by viscoplastic deformation or by recrystallization in the presence of brine. It was also mentioned that, for both plastic and indurated clays, changes in stress state, dilatancy, swelling, and newly formed minerals all might play a role in healing. These insights can be of considerable importance for the investigation of cementitious composites and materials. 

Because a high ability to retard the radionuclide migration is required for the HSULPC, a detailed investigation of the sealing of cracks and pores in water is of considerable importance. It is thus important to observe the detailed sealing process of cracks and pores directly and to clarify the temporal behavior of the sealing. Micro computed tomography (CT) coupled with image analysis technique has been applied to concrete, limestone and sandstone samples before and after bacterial weathering (e.g., [22]) and shown to be quite an effective tool to 3-dimentionally observe the micro structures of the material in a nondestructive manner. However, micro X-ray CT was not applied to HSULPC. For this purpose, Fukuda* et al.* [17] applied micro-focus X-ray CT to observe the sealing process in detail. Specifically, the sealing of a macro-fractured HSULPC specimen with initial fracture width of approximately 0.10 mm was investigated by maintaining the specimen in simulated seawater. The sealing of the macro-fracture occurred only around the outermost parts of the fractured surface of the specimen, and significant precipitation occurred approximately 0.05 mm from the outermost parts of the fractured surface. However, the influence of fracture width, which could be one of the significant factors affecting sealing behavior, has not been clarified yet. 

In this study, the sealing behavior of macro-fractures with different widths in HSULPC in seawater was investigated using micro-focus X-ray CT to clarify the influence of fracture width on fracture sealing in HSULPC.

## 2. Sample Material

As the HSULPC material, “Ductal”, made by Taiheiyo Consultant Co., Ltd. (Sakura, Japan), was used in this study. The composition of the HSULPC specimen is shown in Table 1. The HSULPC specimen was prepared in the way that total composition of the low-heat Portland cement, silica fume, fillers and aggregates became 2300 kg/m^3^ where each component took the range of value in the table. The procedure for the preparation of HSULPC was based on the recommendation proposed by Japan Society of Civil Engineers [23]. The porosity of HSULPC measured by mercury porosimetry was 5% [1,24]. The water permeability coefficient was 4 × 10^−19^ m/s [1,24]. The water to cement ratio was small and ranged from 18%–24%. Other information on the material properties is reported in [1,7,24] in detail. 

**Figure 1:**
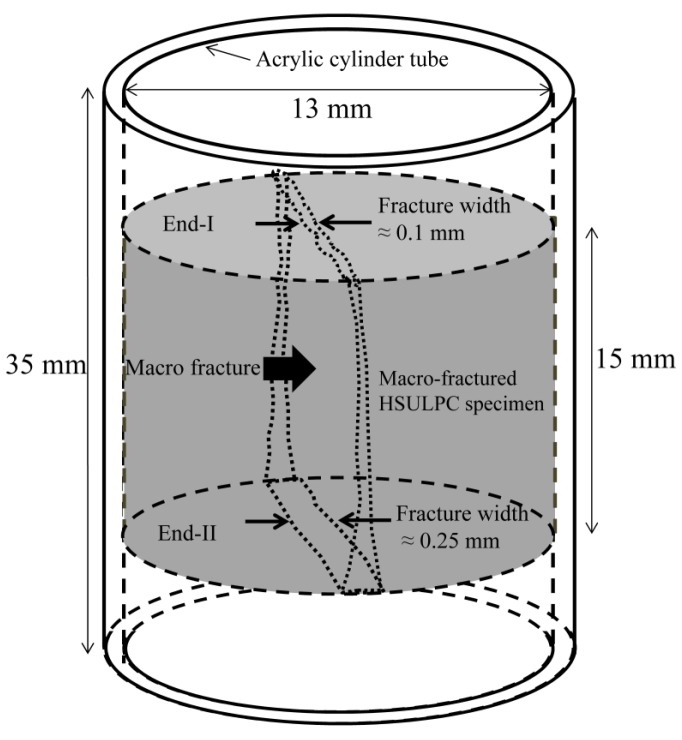
Schematic of macro-fractured HSULPC specimen.

Figure 1 shows a schematic illustration of the macro-fractured HSULPC specimen prepared for this study. This sample was prepared by following the same way in Fukuda* et al.* [17]. An initially intact cylindrical specimen of HSULPC with the diameter and height of approximately 13 and 15 mm, respectively, was split in half axially using a diametrical compression test (also known as diametrical tensile test, Brazilian disk test, indirect tensile test, compact crushing test, or compact hardness test) [25,26], and was set in an acrylic cylinder tube with the height of approximately 35 mm using epoxy glue. To keep the induced fracture open between the induced fracture planes, a thin stainless steel plate with the thickness of 0.10 mm was inserted. Consequently, the initial fracture widths for each outermost part of the fractured surface of specimen became approximately 0.10 mm and 0.25 mm. The terms “End-I” and “End-II” are used to describe the ends with fracture widths of 0.10 and 0.25 mm, respectively. The fracture width inside the specimen gradually increased from End-I to End-II according to preliminary observations by X-ray CT. The age of the HSULPC specimen used in this study was approximately two years. To minimize the errors regarding the water under environmental conditions and scanning position, comparison was conducted within one HSUPLC specimen.

**Table 1:** Composition of high-strength and ultra-low-permeability concrete (HSULPC).

Composition	Amount [kg/m^3^]
Pre-mixed powder	2300 (Note) In case of following the recommendation proposed by Japan Society of Civil Engineers [23], Low-heat Portland cement: 744–1014 Silica fume: 158–496 Fillers: 225–541 Aggregates: 631–947
Water-reducing admixture	24
Water	180

## 3. Observation by X-ray CT

### 3.1. Observation Method 

Salt water is often found underground [27,28,29] and based on the groundwater conditions in Japan, the HSULPC specimen was maintained in simulated seawater in a vacuum (chemical composition in Table 2 [17]). Simulated seawater filled the entire macro-fracture. The mass of water used was 10 times that of the HSULPC specimen. To avoid undesirable dissolution of carbon dioxide in the water, the air in the plastic bottle was replaced with nitrogen. Thus, the plastic bottle was filled with seawater and nitrogen. The sample was placed vertically in the bottle. The specimen was maintained in a thermostatic chamber at 293 K. Because X-rays are more or less attenuated by the presence of water and this causes an undesirable error in the image analysis as discussed below, the HSULPC specimen was dried for a day at room temperature before each X-ray CT observation to remove water from the specimen. Observations were conducted at the initial stage and after maintaining the specimen in water for 21 and 49 days. The same seawater was used for the entire observation period. Because the amount of the simulated seawater decreased little when the sample was put out from the plastic bottle, the seawater was refilled before each immersion.

**Table 2:** Chemical composition of simulated seawater (mol L^−1^) (Adapted from Fukuda* et al.* [17]).

Ca^2+^	SO_3_^2−^	Na^+^	K^+^	Cl^−^	Mg^2+^	HCO_3_^−^
10 × 10^−3^	29 × 10^−3^	45 × 10^−2^	19 × 10^−3^	56 × 10^−2^	55 × 10^−3^	24 × 10^−4^

A micro-focus X-ray CT scanner, TOSCANER 31300 μhd (Toshiba IT & Control Systems Co., Ltd., Tokyo, Japan), installed at Hokkaido University, Japan, was used [17,30,31]. The device is considered to be a micro-CT scanner [32] with maximum spatial resolution of 5 μm. The detailed methodology of the X-ray CT observation is described in Fukuda* et al.* [17].

In total, 680 CT slice images were obtained to reconstruct the entire specimen and to identify the precipitated regions in this study.

### 3.2. Results 

**Figure 2:**
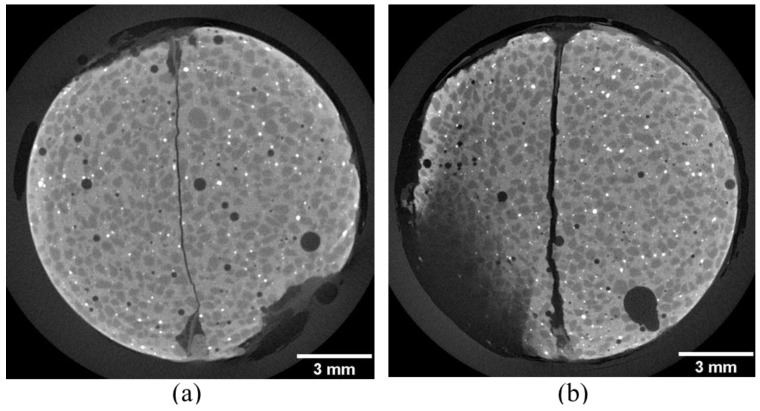
X-ray CT slice images of macro-fractured HSULPC specimen at the initial stage (**a**) near End-I and (**b**) near End-II.

Typical CT slice images near End-I and End-II of the specimen in the initial stage are shown in Figure 2. The cross sections also correspond to the tops in the image analysis described in the next section. These images are presented in gray-scale with a range of 256 shades from black to white. High CT values, shown in white or brighter colors, correspond to regions with higher density. Regions of low CT values, shown in black or darker colors, correspond to areas of lower density. The straight black lines through the specimen found in the central part of each image are the macro-fractures with approximate widths of 0.10 and 0.25 mm, respectively. The dark circular shapes of various sizes are pores, with approximately spherical shapes in the three-dimensional sense. Small white granular regions having higher density are also found over the whole slices of End-I and End-II,* i.e.*, over the whole specimen, and these correspond to metal included in the silica fume as a byproduct of metal refining. The other regions, shown in gray, are the HSULPC matrix and consist of cement and aggregates. 

Most of the pores with diameters greater than ~20 μm were isolated from one another, and some of the pores were on the specimen surface. However, the pore connectivity of much smaller size (<1 μm) was not visible because of the aforementioned CT image resolution.

**Figure 3:**
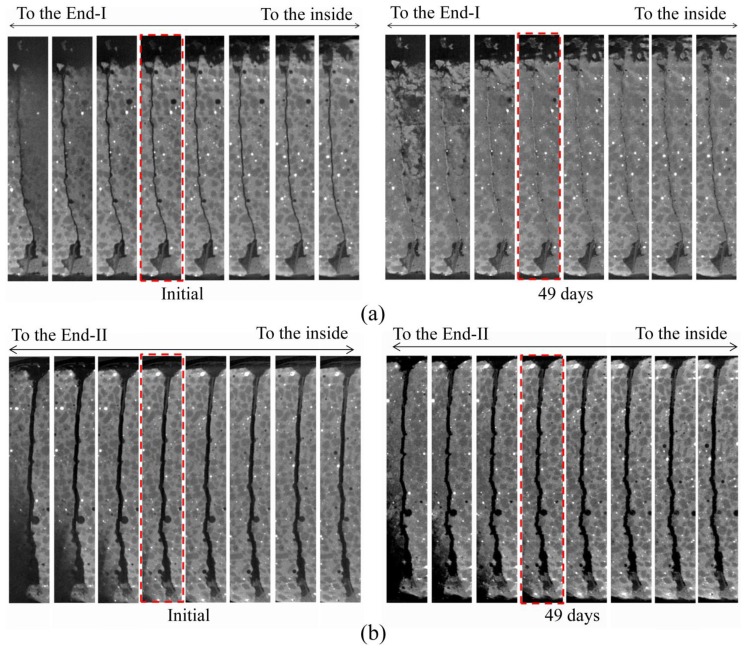
Comparison of CT images of macro-fracture at initial stage and after being kept in seawater for 49 days for (**a**) End-I and (**b**) End-II. Height and width of each image are 14 and 2 mm, respectively. Note that the red dashed lines correspond to the top cross sections of each region of interest (ROI) in Figure 5.

Figure 3 shows CT images of the macro-fracture for approximately the same cross sections near End-I and End-II, respectively. These images were extracted from seven CT images of the specimen at intervals of 24 μm from each end to the interior of the specimen, at the initial stage and after being kept in seawater for 49 days. In the region close to End-I, most parts of the macro-fracture were sealed in the specimen kept in seawater for 49 days, and less sealing was observed towards the specimen interior. This result shows a similar tendency reported by Fukuda* et al.* [17] where the macro-fracture of the HSULPC was approximately 0.10 mm. In the region close to End-II, although precipitation occurred in the macro-fracture in the specimen kept in seawater for 49 days, sealing was not observed. In addition, less precipitation was found towards the specimen interior. Note that the CT images nearest both ends showed more or less blurring because of the partial volume effect (e.g., [32]), where voxels are affected by including both solid parts and air within them. Such blurring is also caused by the tilted surface of the specimen at both ends.

**Figure 4:**
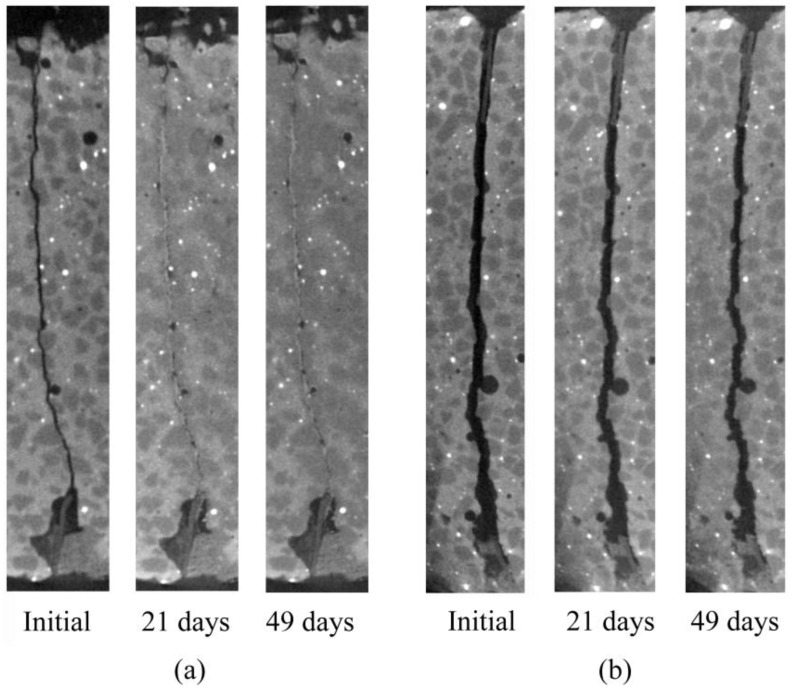
Comparison of CT images for a particular section near (**a**) End-I and (**b**) End-II. Height and width of each image are 14 and 2 mm, respectively.

Figure 4 shows the temporal change in precipitation on the macro-fracture for one cross section near End-I and End-II, respectively. These cross sections are approximately the same sections near each end as those in Figure 2, and are also those indicated by dotted lines in Figure 3. In Figure 4a, precipitation occurred when the specimen was kept in water for 21 days and the sealed area increased with elapsed time. Because no re-opening of the sealed crack was found, we did not consider that dissolution of the precipitates occurred over the period of observation. No swelling of the specimen was found during the period of observation. In Figure 4b, a smaller amount of precipitation compared with that in Figure 4a was found when the specimen was kept in water for 21 days, and the precipitated area increased slightly with elapsed time, although significant sealing was not observed when the specimen was kept in water for 49 days.

The following observations were clarified by X-ray CT. Precipitation occurred at End-I and End-II with different fracture widths to the interior of the specimen within a range of approximately 0.3 mm. The amount of precipitate increased with elapsed time closer to each end. Thus, significant sealing was attained only in the macro-fracture close to End-I and sealing could be attained only in the case of the smaller fracture width. 

Near End-I, most parts of the macro-fracture were sealed after 21 days because of precipitation. Once such sealing was achieved, little precipitation was observed in the macro-fracture below the sealed part. For End-II, it seems that precipitation in the macro-fracture was still in progress after 49 days. In addition, in terms of the influence of fracture width on the attainment of sealing, although the considered cementitious material was different, the results obtained in the HSULPC showed a similar tendency in the self-sealing of engineered cementitious composites reported by Yang* et al.* [9].

Based on these results, image analyses were conducted to evaluate the detailed sealing process only near End-I and End-II of the specimen, where precipitation was observed in the macro-fracture.

## 4. Image Analysis

### 4.1. Image Processing Method 

To investigate temporal changes in the precipitated regions of the macro-fracture, it was necessary to extract the corresponding regions by image processing. For the CT images where precipitation was observed, we sought to extract precipitates by applying an image subtraction technique between the CT images at the initial stage and after the specimen was kept in water. However, because of the need to set the specimen on the table in the CT scanner manually before each scan, alignment gaps between the comparison images were inevitable and image registration was necessary.

**Figure 5:**
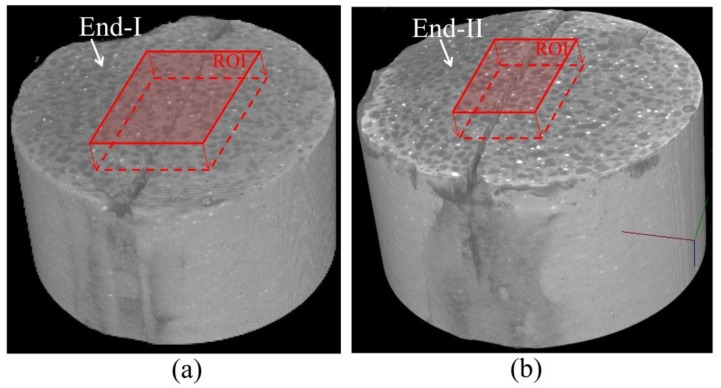
3D CT image of (**a**) upper and (**b**) lower halves of the HSULPC specimen used in image analysis, where the ROIs used in the image registration are indicated by rectangular parallelepipeds. Height, width and depth of each parallelepiped are 4.3 mm × 4.8 mm × 0.7 mm and 2.9 mm × 4.5 mm × 0.7 mm, respectively. The rectangles shown by red solid lines are on the cross section of each end of the specimen and red arrows indicate the depth toward other end of the specimen.

This research applied the same image registration approach used by Fukuda* et al.* [17] and a complete description of the approach is provided there. Briefly, CT images obtained after keeping the specimen in water were mapped onto those at the initial stage through an affine transform. To estimate the transform coefficients, the least square method was applied using gaps found in multiple sampling points represented by white granular regions. Then, the transform was optimized by utilizing the maximum value of the volume correlation between comparison images in the particular regions of interest (ROIs). The shapes of the ROIs for End-I and End-II were set as rectangular parallelepipeds (Figure 5), where the ROIs are presented by red lines with the 3D images of the upper and lower halves of the HSULPC specimen. The thicknesses of both the ROIs are equivalent to 30 slices from each end to the interior of the specimen. Considering the precision of the image subtraction, the cross sectional matrix sizes of each ROI were 270 × 300 and 180 × 280 pixels, respectively. Because the nearest cross section to each end results in blurred CT images and can cause significant error in the image analyses, blurred CT images were excluded from the top of each ROI. Consequently, the top cross sections of each ROI are those surrounded by dashed lines in Figure 3. Following the image registration, image subtraction was conducted for each end.

### 4.2. Results

**Figure 6:**
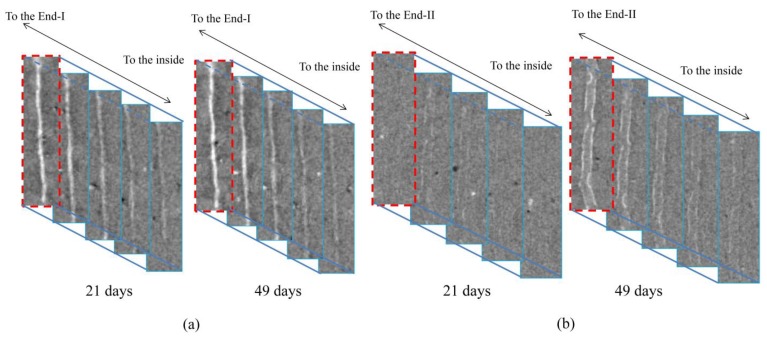
Subtraction images between initial images and those obtained after specimen was kept in seawater for 21 or 49 days for (**a**) End-I and (**b**) End-II. Height and width of each subtraction image are (**a**) 2.9 and 0.7 mm; and (**b**) 3.5 and 0.9 mm, respectively.

Figure 6 shows results of the image subtraction process (*i.e.*, subtraction images) in each ROI in Figure 5. In the image subtraction process, the reference images correspond to the specimen at the initial stage, and comparison images correspond to the specimen after 21 or 49 days in seawater. In Figure 6, the images surrounded by red dotted lines correspond to the same plane for each time. From these images toward the inside of the specimen, the images surrounded by blue dotted lines show the same planes, respectively, for each time. In the subtraction images, only the macro-fractures in each ROI are presented at an interval of 48 μm from the top of each end to the interior of the specimen. These images are displayed in gray-scale, with a range of 256 shades from black to white, for a window level and width of 0 and 1000, respectively. In regions where no change occurred, the differences between the CT values are approximately zero. In this case, images are displayed in gray. In regions where precipitation occurred, the difference between the CT values is positive. In this case, the color is white. From Figure 6, the regions of precipitation observed in Figure 3 are extracted successfully from each macro-fracture.

**Figure 7:**
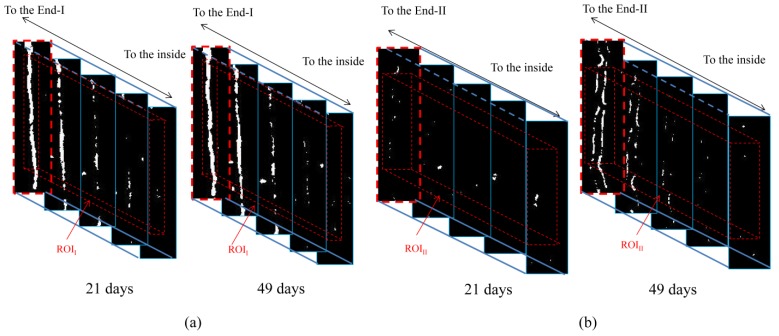
Binarized images obtained by segmentation of subtraction images in Figure 6 for (**a**) End-I and (**b**) End-II. Height and width of each subtraction image are (**a**) 2.9 and 0.7 mm; and (**b**) 3.5 and 0.9 mm, respectively.

In the subtraction images, reasonable segmentation between precipitated and non-precipitated regions was required. If a voxel [32] includes both the precipitated and non-precipitated regions, the CT value of this voxel takes an intermediate value between these two phases. This kind of voxel is called a “mixel” [33,34,35]. A maximum likelihood thresholding method considering the effect of mixels [33,34] was used to set an appropriate threshold. The calculation of threshold in Figure 6 was based on exactly the same procedure described in Fukuda* et al.* [17] using a histogram of the probability density function in each ROI of the subtraction image. Binarized images obtained from the subtraction images in Figure 6 by the maximum likelihood thresholding method are shown in Figure 7. The images show the cross sections from the top to the interior of each ROI at an interval of 48 μm, and demonstrate the increase in amount of precipitation with elapsed time successfully.

## 5. Discussion 

The X-ray CT observation results in Section 3 showed that sealing of the macro-fracture by precipitation occurred only near both ends of the macro-fracture in the specimen with different fracture widths. Because the age of the HSULPC specimen used in this study was approximately 2 years, it is sufficiently old and it could be highly possible that the primary mechanism for the precipitation is calcium carbonate [36,37]. This suggests that, the macro-fracture near the ends was located in an environment where the precipitation of calcium compounds consisting mainly of calcium carbonate was enhanced by the dissolution of calcium ions from the HSULPC and both the carbonate and bicarbonate ions found in the seawater [36,37] although this may not be the case if flow,* i.e.*, a pressure gradient was encouraged through the sample. Hence the cement hydration products dissolved in water, and then the liberation and dissipation of the calcium hydroxide could occur along the fracture surfaces. Free calcium ions from cement hydration or the surrounding water environment then reacted with dissolved carbon dioxide, forming crystals of calcium carbonate which grew at both fracture surfaces [5]. This can be explained by the relatively high concentration of calcium hydroxide near the crack surface [36,37] through the diffusion process from the bulk cementitious material and complex fractal surface, which can serve as a calcite nucleation site [8]. This view is supported by the fact that precipitated calcium carbonate was observed at the outside surfaces of the fracture as a white residue in the HSULPC specimens used in this study and by Fukuda* et al.* [17], and was also reported in various cementitious materials (e.g., [4]).

If the macro-fracture is sealed near the end, the network of cracks inside the HSULPC can be isolated from the surrounding environment, which can cause a decrease in permeability [38] and retard degradation in terms of the ability to retard the radionuclide migration of the HSULPC. Because CT observations suggest that the amount of precipitated regions increases with elapsed time, an investigation of the temporal change of the sealing deposits and an evaluation of the effect of precipitates on limiting water flow into the HSUPLC could be of engineering interest. Thus, it is valuable to evaluate the temporal change of the sealing deposits in the macro-fracture.

**Figure 8:**
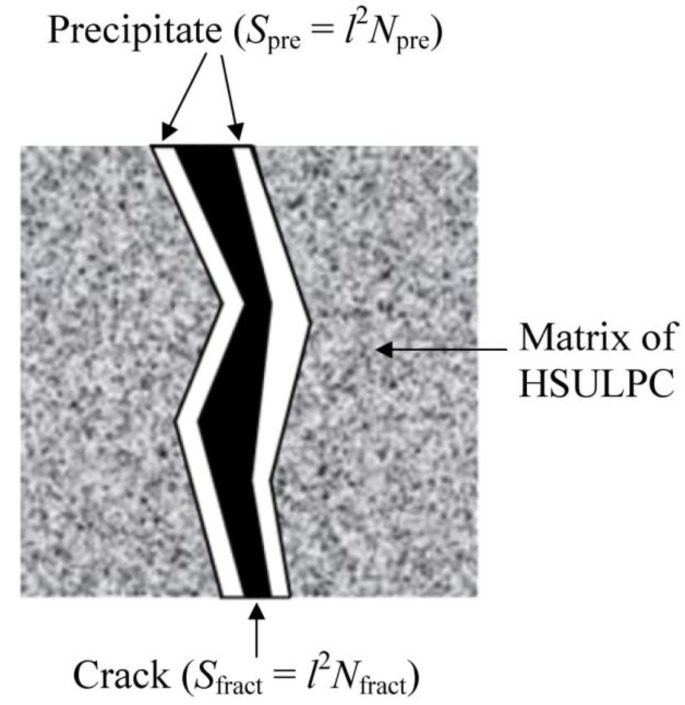
Schematic diagram of precipitation on the fracture surface in each slice.

The 30 binarized images obtained in the previous section were used to evaluate the precipitation process, especially in ROI_I_ and ROI_II_ in Figure 7. Figure 8 shows a schematic illustration of precipitation in the macro-fracture. The percentages of sealing deposits in the macro-fracture, *P*_seal_ (%), in each slice, having slice thickness *T*_slice_, were calculated:

(1) $$P_{\text{seal}}=\frac{T_{\text{slice}}S_{\text{pre}}}{T_{\text{slice}}S_{\text{fract}}}\times 100=\frac{l^{2}N_{\text{pre}}}{l^{2}N_{\text{fract}}}\times 100=\frac{N_{\text{pre}}}{N_{\text{fract}}}\times 100\left(\%\right)$$

where *S*_pre_ (μm^2^) is the total precipitate area, *S*_fract_ (μm^2^) is the total area of the macro-fracture, *l* (μm) is the pixel size (16 μm), and *N*_pre_ and *N*_fract_ are the number of pixels for precipitates and the macro-fracture, respectively. The *N*_pre_ in each ROI was obtained from the extracted precipitates in the binarized images. *N*_fract_ was obtained by counting the number of pixels corresponding to the macro-fractures in each ROI by applying the maximum likelihood thresholding method to the CT images at the initial stage, where the regions of the macro-fracture and matrix of the HSULPC were segmented. Because the mean values of the fracture widths in each ROI were approximately constant,* i.e.*, 0.08 and 0.25 mm, respectively, the temporal changes in *P*_seal_, denoted as *R*_seal_ (% day^−1^), were computed using:

(2) $$R_{\text{seal}}=\frac{\Delta P_{\text{seal}}}{\Delta t}\left(\%\cdot {\text{day}}^{-1}\right)$$

where Δ*P*_seal_ (%) is the change in *P*_seal_ over a given immersion period, Δ*t* (day).

**Figure 9:**
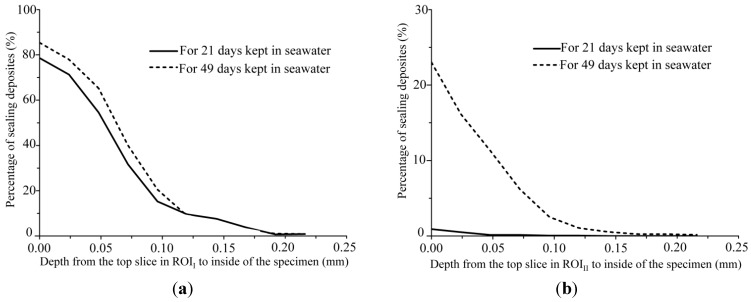
Relationship between percentage and position of sealing deposits in specimen kept in seawater for 21 and 49 days for (**a**) ROI_I_ and (**b**) ROI_I__I_.

*P*_seal_ in each ROI is shown in Figure 9 with respect to the depth from the top to the interior of each ROI. In Figure 9, Δ*t* was 21 days if taken 21 days after placing the specimen in water. Similarly, Δ*t* was 28 days if the specimen was placed in water at 22 days and taken out of the water at 49 days. From these figures, *P*_seal_ increased towards both ends in each period, and *P*_seal_ at a depth of 0.2 mm for both ROIs increased with elapsed time. If the thickness of the excluded blurring images in each ROI corresponds to approximately 0.10 mm mentioned in Section 3 is considered, precipitations for both ends were found within a depth of 0.3 mm from each end to the interior of the specimen. In addition, considering the macro fracture of unit fracture length for the tops of ROI_I_ and ROI_II_, Figure 9 shows that the values of *P*_seal_ for each ROI are largely different from each other. However, as shown in Table 3, maximum amounts of precipitation at 49 days for the tops of each ROI are roughly the same. Thus, if it can be assumed that the expected amount of precipitation is determined by the composition of HSUPLC and surrounding water and that such amount is approximately given by that at the top in ROI_I_, further significant sealing in End II could not be expected because the expected amount of precipitation has been almost attained at 49 days.

**Figure 10:**
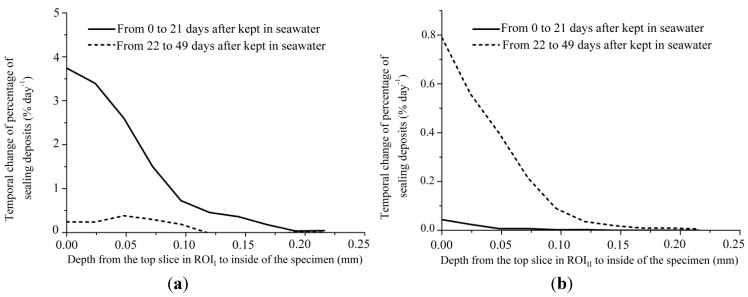
Temporal change of percentage of sealing deposits for specimen kept in seawater for 0–21 days and 22–49 days in (**a**) ROI_I_ and (**b**) ROI_I__I_.

Similarly, *R*_seal_ in each ROI is shown in Figure 10 with respect to the depth from the top to the interior of each ROI. From Figure 10, *R*_seal_ towards each end tends to be larger than that in the inner region. In ROI_I_, the maximum *R*_seal_ was found at 21 days, and then *R*_seal_ decreased. It is also suggested that little precipitation could be expected to occur after 49 days in ROI_I_, and, once the macro-fracture is almost sealed, less precipitation could be expected in the region below the sealed parts. This tendency supports the view that the sealing of macro-fractured HSULPC specimens with fracture width of 0.10 mm occurs by 21 days in seawater as noted by Fukuda* et al.* [17]. On the other hand, in ROI_II_, the maximum *R*_seal_ at 49 days was larger than that at 21 days. Thus, although further precipitation after 49 days may occur in ROI_II_, considering the above discussion regarding *P*_seal_, the required time for the sealing of a fracture in ROI_II_ will be much longer. The difference of *R*_seal_ in each ROI was caused by the difference in fracture width and greater calcite nucleation was enhanced in the smaller fracture width.

**Table 3:** Maximum amount of precipitation on macro fracture at 49 days for each end.

	Position	*P*_seal_ (%)	Mean fracture width, *W* (mm)	Fracture length, *L* (mm)	Area of precipitation calculated by *P*_seal_ × *W* × *L* (mm^2^)
End I	Top in ROI_I_	85	0.08	1	0.068
End II	Top in ROI_II_	23	0.25	1	0.058

Based on the above discussion, when HSULPC is kept in seawater, precipitation of calcium compounds occurs in the fracture near the end, and the sealing deposits increase with elapsed time. Considering the application of HSULPC, it is desirable that such sealing should occur over a short period and, in terms of optimum fracture width, the self-sealing of the fracture can occur for a fracture with width of 0.10 mm or less in the case of HSULPC. In addition, identifying optimum water conditions and water pressures are of considerable importance. To clarify these conditions, a more detailed investigation regarding the influence of water conditions on sealing and the change in water content because of precipitation could also be important. This is beyond the scope of this study, and we regard it as future work.

## 6. Conclusions

The sealing process of a macro-fracture in HSULPC in simulated seawater was investigated in this study. A macro-fractured HSULPC specimen with different fracture widths of 0.10 and 0.25 mm at each outermost part of the fractured surface was prepared and kept in simulated seawater for up to 49 days. The surface and interior of the specimen were then observed using micro-focus X-ray CT.

Significant precipitation occurred approximately 0.3 mm from each outermost part of the fractured surface. Temporal changes in sealing in the macro-fracture showed that the sealing deposits near each outermost part of the fractured surface increased with time if the specimen was kept in water for a longer period. Additionally, the sealing deposits increased towards each outermost part of the fractured surface of the HSULPC specimen. The time at which maximum precipitation rate was attained depended on fracture width. The significant sealing was only attained for the 0.10 mm fracture width,* i.e.*, approximately 85% in terms of maximum percentage of sealing deposits in the specimen within the observation period (49 days). On the other hand, sealing was not attained within the observation period in the case of 0.25 mm fracture width where maximum percentage of sealing deposits in the specimen was approximately 23%. For the rate of temporal change of percentage of sealing deposits, the maximum rate, approximately 3.75%·day^−1^, was attained in case of 0.10 mm fracture width at 21 days, and it decreased with time. On the other hand, in case of 0.25 mm fracture width, the rate was found to increase within the observation period and the approximately 0.8%·day^−1^ at 49 days was attained. 

Therefore, the difference in fracture width of the HSULPC was found to be a critical factor for the attainment of self-sealing of the fracture. Results obtained in this paper are important for the geological disposal of radioactive wastes, and further accumulation of relevant information is important.
